# Mutual Effects of Orexin and Bone Morphogenetic Proteins on Catecholamine Regulation Using Adrenomedullary Cells

**DOI:** 10.3390/ijms25031585

**Published:** 2024-01-27

**Authors:** Yoshiaki Soejima, Nahoko Iwata, Koichiro Yamamoto, Atsuhito Suyama, Yasuhiro Nakano, Fumio Otsuka

**Affiliations:** Department of General Medicine, Okayama University Graduate School of Medicine, Dentistry and Pharmaceutical Sciences, 2-5-1 Shikata-cho, Kitaku, Okayama 700-8558, Japan; p32v0ja8@s.okayama-u.ac.jp (Y.S.); asuyama@s.okayama-u.ac.jp (A.S.); y-nakano@okayama-u.ac.jp (Y.N.)

**Keywords:** bone morphogenetic protein (BMP), orexin, catecholamine and adrenal

## Abstract

Orexins are neuronal peptides that play a prominent role in sleep behavior and feeding behavior in the central nervous system, though their receptors also exist in peripheral organs, including the adrenal gland. In this study, the effects of orexins on catecholamine synthesis in the rat adrenomedullary cell line PC12 were investigated by focusing on their interaction with the adrenomedullary bone morphogenetic protein (BMP)-4. Orexin A treatment reduced the mRNA levels of key enzymes for catecholamine synthesis, including tyrosine hydroxylase (Th), 3,4-dihydroxyphenylalanie decarboxylase (Ddc) and dopamine β-hydroxylase (Dbh), in a concentration-dependent manner. On the other hand, treatment with BMP-4 suppressed the expression of Th and Ddc but enhanced that of Dbh with or without co-treatment with orexin A. Of note, orexin A augmented BMP-receptor signaling detected by the phosphorylation of Smad1/5/9 through the suppression of inhibitory Smad6/7 and the upregulation of BMP type-II receptor (BMPRII). Furthermore, treatment with BMP-4 upregulated the mRNA levels of OX1R in PC12 cells. Collectively, the results indicate that orexin and BMP-4 suppress adrenomedullary catecholamine synthesis by mutually upregulating the pathway of each other in adrenomedullary cells.

## 1. Introduction

Orexins are neuronal peptides that play a prominent role in sleep behavior, feeding behavior, autonomic function and stress response in the central nervous system [1]. Orexin A and orexin B are enzymatic cleavage products of a precursor, prepro-orexin and function through G protein-coupled receptors, orexin receptor type 1 (OX1R) and orexin receptor type 2 (OX2R) [2]. In addition to the central nervous system, functions of orexins and orexin receptors have been discovered in peripheral tissues, including the intestine, pancreas, adipose tissue, gonads, cardiovascular system and adrenal gland [3,4]. It has been reported that orexins function in intestinal motility, insulin and glucagon secretion, lipid metabolism, testicular androgen production, ovulation, the regulation of blood pressure, adrenocortical steroidogenesis and adrenomedullary catecholamine synthesis [3].

In the adrenal gland, orexins have functions in the regulation of steroidogenesis in the cortex and catecholamine synthesis in the medulla [4,5,6]. Orexins stimulate glucocorticoid and mineralocorticoid secretion from the adrenal cortex in various species [7,8,9,10]. Moreover, it was reported that orexins enhanced the secretion of basal but not adrenocorticotropin (ACTH)-stimulated glucocorticoid and that an antagonist of the ACTH receptor attenuated the glucocorticoid response to ACTH but not that of orexin [11]. Thus, it has been shown that orexins stimulate the secretion of adrenal steroids both directly and via the hypothalamic–pituitary–adrenal (HPA) axis.

Studies on the possible functions of orexins in the adrenal medulla have been limited compared to studies in the adrenal cortex. It was reported that both OX1R and OX2R are present in both the rat and human adrenal medulla [11,12]. Nanmoku et al. reported that orexin suppresses the expression of tyrosine hydroxylase (Th), the rate-limiting enzyme in the biosynthesis of catecholamine, and dopamine secretion in rat pheochromocytoma PC12 cells [13]. These results indicate regulatory functions of orexin in the adrenal medulla, as well as in the adrenal cortex. However, the mechanisms underlying the functions of orexin in the adrenal medulla have not been elucidated.

There has been an accumulation of evidence indicating that bone morphogenetic proteins (BMPs), members of the transforming growth factor (TGF)-β superfamily, have functional roles in various endocrine organs [14]. Among these, the presence of a local BMP system in the adrenal medulla [15,16], as well as in the adrenal cortex [17], has been revealed. In our earlier studies, the expression of BMPs and BMP receptors was detected in PC12 cells [15]. We reported that BMP-2, BMP-4, BMP-7 and activin were expressed in PC12 cells and reduced the secretion of dopamine [15]. Of note, among the BMP ligands, BMP-4 preferentially enhanced the aldosterone-induced mRNA expression of tyrosine hydroxylase (Th) [16]. We also showed that melatonin, a pineal gland hormone that plays an important role in sleep regulation and circadian functions [18], modulates catecholamine biosynthesis by interacting with glucocorticoids and BMP-4 [19]. However, the functional inter-relationship between orexin and adrenomedullary BMPs has not been investigated.

The aim of this study was to determine the functional roles of orexin in the regulation of adrenomedullary catecholamine biosynthesis, with a focus on interaction with the BMP system in the adrenal medulla.

## 2. Results

**Figure 1:**
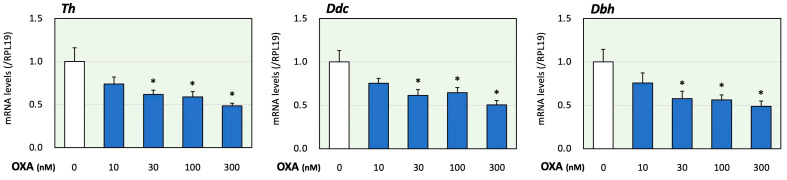
Effects of orexin A on the expression of key enzymes for catecholamine biosynthesis in adrenomedullary cells. PC12 cells (3 × 10^5^ cells/mL) were treated with orexin A (OXA; 10–300 nM) in DMEM containing 1% FBS and 1% HS for 24 h. Next, total cellular RNAs were extracted, and the mRNA levels of Th, Ddc and Dbh were determined. The expression levels were standardized using RPL19 mRNA levels and expressed as fold changes (n = 9). The results are shown as means ± SEM and were analyzed using an ANOVA with Tukey–Kramer’s post hoc test; *, *p* < 0.05 vs. the control group.

First, the effects of orexin on adrenomedullary catecholamine biosynthesis were evaluated. In the present study, we examined the effects of orexin A since orexin A, but not orexin B acts via both OX1R and OX2R [1]. The treatment time of orexin A was determined according to a previous study using adrenocortical cells [17]. In this study, using PC12 cells that express catecholamine-producing enzymes [20], the expression levels of key enzymes for catecholamine biosynthesis were evaluated. Tyrosine was converted to DOPA via the action of Th. DOPA was then converted to dopamine using Ddc. Dbh converted dopamine to noradrenaline [20]. It was reported that the expression levels of the synthetic enzymes Th, Ddc and Dbh were all similar to or higher than those found in the adrenal glands [20]. As shown in Figure 1, treatment with orexin A (10 to 300 nM) for 24 h reduced the mRNA levels of Th, Ddc and Dbh in a concentration-dependent manner. Based on these results and the results obtained in a previous study [13], the orexin A concentration was fixed to 100 nM in the following experiments.

**Figure 2:**
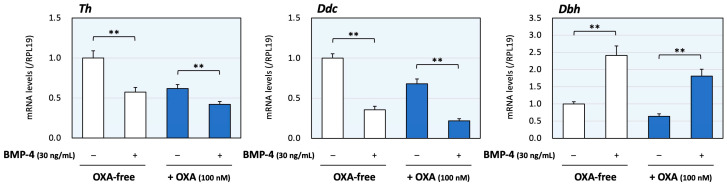
The effects of orexin A and BMP on the expression of enzymes during catecholamine synthesis in adrenomedullary cells. PC12 cells (3 × 10^5^ cells/mL) were treated with BMP-4 (30 ng/mL) and/or orexin A (OXA; 100 nM) in DMEM containing 1% FBS and 1% HS for 24 h. Total cellular RNAs were extracted, and the mRNA levels of Th, Ddc and Dbh were standardized using RPL19 mRNA levels and expressed as fold changes (n = 9). The results are shown as means ± SEM and were analyzed using the unpaired *t*-test; **, *p* < 0.01 between the indicated groups.

Next, the effects of co-treatment with orexin A and BMP-4 were evaluated. The dose of BMP-4 was determined from our previous study [16]. As shown in Figure 2, BMP-4 (30 ng/mL) treatment decreased the mRNA levels of Th and Ddc, although the expression levels of Dbh were increased by BMP-4 treatment. In the presence of orexin A (100 nM), BMP-4 treatment also reduced the expression levels of Th and Ddc. On the other hand, BMP-4 increased Dbh mRNA levels regardless of the presence of orexin A, which can reduce Dbh expression. Thus, the treatment with BMP-4 acted to regulate the expression of Th and Ddc collaborating with orexin A, whereas BMP-4 counteractively enhanced the expression of Dbh in the presence of orexin A.

**Figure 3:**
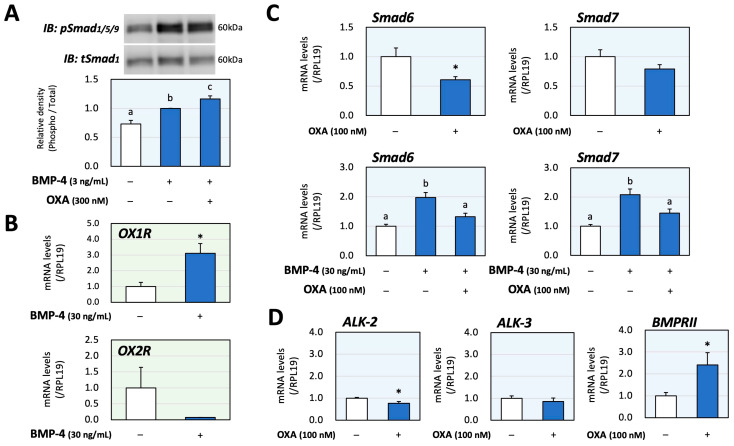
Interactions between orexin A and BMP during catecholamine biosynthesis via PC12 cells. (**A**) PC12 cells (3 × 10^5^ cells/mL) were pretreated with orexin A (OXA; 300 nM) in DMEM containing 1% FBS and 1% HS for 48 h and were then stimulated with BMP-4 (3 ng/mL) for 1 h. The cells were lysed and then subjected to immunoblot (IB) analysis using anti-pSmad1/5/9 and anti-tSmad1 antibodies. The integrated signal density of each protein band was digitally analyzed, and the ratios of the signal intensities of pSmad/tSmad were calculated. The results are representative of those obtained from at least three independent experiments and are expressed as fold changes (n = 6). (**B**) Cells (3 × 10^5^ cells/mL) were treated with BMP-4 (30 ng/mL) in DMEM containing 1% FBS and 1% HS for 24 h. Total RNAs were extracted, and the mRNA levels of OX1R and OX2R were standardized using RPL19 levels and expressed as fold changes (OX1R, n = 9; OX2R, n = 3). (**C**) Cells (3 × 10^5^ cells/mL) were treated with OXA (100 nM) and/or BMP-4 (30 ng/mL) in DMEM containing 1% FBS and 1% HS for 24 h. Total RNAs were extracted, and the mRNA levels of Smad6 and Smad7 were standardized using RPL19 levels and expressed as fold changes (n = 9). (**D**) Cells (3 × 10^5^ cells/mL) were treated with OXA (100 nM) in DMEM containing 1% FBS and 1% HS for 24 h. Total RNAs were extracted, and the mRNA levels of BMP receptors (ALK-2, -3 and BMPRII) were standardized using RPL19 levels and expressed as fold changes (n = 9). The results are shown as means ± SEM and were analyzed using an ANOVA with Fisher’s PLST test (**A**), Tukey–Kramer’s post hoc test (**C**) or the unpaired *t*-test (**B**–**D**). Values with different superscript letters are significantly different at *p* < 0.05. * *p* < 0.05 vs. the control group.

Next, the interactions of orexin and BMP signaling during catecholamine synthesis were evaluated using PC12 cells. The phosphorylation of Smad1/5/9, which was activated via treatment with BMP-4 (3 ng/mL) for 1 h, was significantly enhanced due to orexin A (300 nM) pretreatment for 48 h according to the results of the Western blot analysis (Figure 3A); the concentrations of BMP-4 and orexin A as well as the treatment time, were optimized to clearly detect the signal intensities. The changes in mRNA expression of OX1R and OX2R caused by BMP-4 were then examined in PC12 cells, in which the basal expression level of OX2R mRNA was lower than that of OX1R mRNA. Treatment with BMP-4 (30 ng/mL) significantly upregulated the expression of OX1R, whereas treatment with BMP-4 tended to decrease the expression of OX2R (Figure 3B). The detailed mechanism by which orexin A enhances BMP signaling in PC12 cells was then examined. As shown in Figure 3C, orexin A (100 nM) significantly reduced the expression of inhibitory Smad6 while not affecting the expression of inhibitory Smad7. Meanwhile, orexin A significantly reduced the expression of both Smad6 and Smad7, which was induced by BMP-4 (30 ng/mL) treatment. Regarding the BMP receptors, orexin A (100 nM) moderately decreased the mRNA levels of ALK-2 (23%) and ALK-3 (15%) but increased the mRNA level of BMPRII (141%) compared with the level in the control group (Figure 3D). These results suggest that orexin A enhances BMP signaling through the suppression of the expression of inhibitory Smads and upregulation of functional BMP receptors.

## 3. Discussion

**Figure 4:**
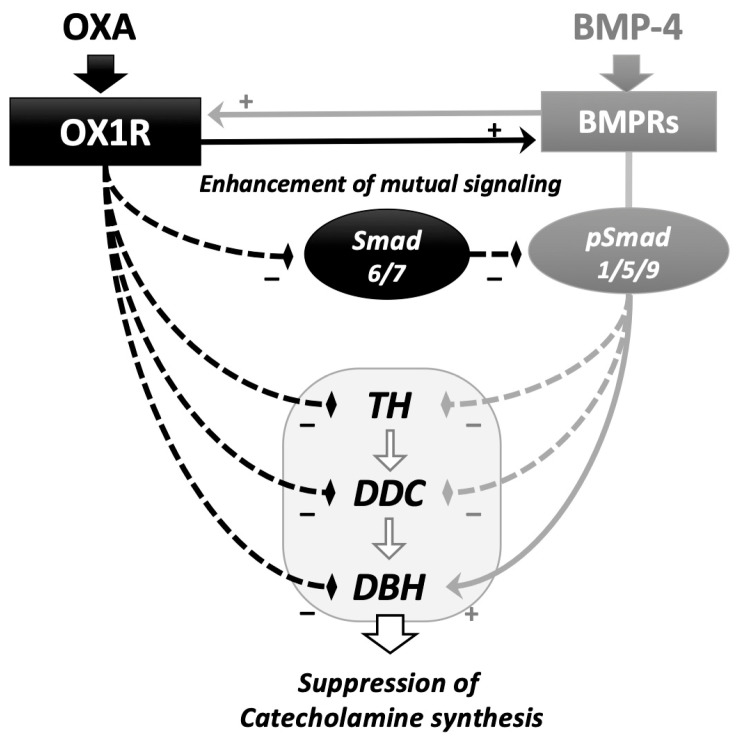
Functional interaction of the signaling between orexin A and BMP in adrenomedullary cells. Orexin A (OXA) suppressed the expression of key enzymes for catecholamine biosynthesis, including Th, Ddc and Dbh. BMP-4 downregulated the expression of Th and Ddc and enhanced the expression of Ddc in the presence and absence of OXA. OXA enhanced BMP-4-induced Smad1/5/9 phosphorylation via the downregulation of the expression of inhibitory Smad6/7 and upregulation of the expression of BMP receptors (BMPRs). BMP-4 upregulated the expression of orexin receptor type I (OX1R). In adrenomedullary cells, functional interactions between the signaling of orexin A and BMP during the regulation of catecholamine biosynthesis were demonstrated.

In this study, the interactions between orexin A and BMP signaling during catecholamine synthesis were revealed in rat adrenomedullary PC12 cells (Figure 4). Orexin A suppressed the expression of the key enzymes during catecholamine biosynthesis, in which BMP-4 decreased the expression of Th and Ddc but increased that of Dbh in the presence and absence of orexin A. Since the amount of noradrenaline secreted from PC12 cells is limited compared to that of dopamine [20], the effects of orexin A and BMP-4 on the expression of Dbh are unclear. However, it was interesting that BMP-4 and orexin A collaboratively suppressed the expression of Th and Ddc, although BMP-4 acted to counteract orexin A and increase the Dbh expression in PC12 cells. Orexin A activated BMP signaling by suppressing the expression of inhibitory Smads and upregulating the expression of BMP receptors. Meanwhile, BMP-4 treatment upregulated the expression of OX1R. These results suggest that orexin and BMP-4 suppress adrenomedullary catecholamine synthesis by mutually upregulating the signaling of each other in PC12 cells.

In the present study, orexin A reduced the expression of rate-limiting enzymes during catecholamine biosynthesis (Figure 1). These results are consistent with the results of the study by Nanmoku et al., showing that both orexin A and orexin B reduced Th mRNA levels, cAMP levels and dopamine secretion in a dose-dependent fashion [13]. A study using Sprague Dawley rats and PC12 cells overexpressing OX1R showed that orexin stimulates calcium/calmodulin-dependent kinase II via OX1R activation and calcium influx [21]. In cultured rat adrenomedullary cells, it was shown that orexin A triggers catecholamine release through the activation of OX1R and the extracellular calcium influx [22]. These results indicate that the orexin system of rats is involved in the regulation of catecholamine synthesis and release via OX1R and calcium influx, although this action is dependent on the cell line. Since the PC12 cells used in our experiment were obtained from a rat pheochromocytoma, the reaction against orexin stimulation might be different from that in physiological in vivo studies.

Moreover, it seems that the effects of orexin on adrenomedullary catecholamine synthesis are different among species. A study using cultured bovine adrenomedullary cells demonstrated that orexin A (0.1 nM) enhanced Th activity and catecholamine synthesis via OX1R coupled to the protein kinase C (PKC) pathway [23]. A study by Nemoto et al. showed that OX1R, OX2R and prepro-orexin were expressed in bovine adrenomedullary cells. Both orexin A and orexin B induced intracellular calcium changes and stimulated adrenaline release and these effects were blocked by treatment with an orexin antagonist. Moreover, orexin treatment decreased the expression of OX1R, OX2R and prepro-orexin [24]. These results indicate that the orexin system is involved in the regulation of catecholamine synthesis and release via the PKC pathway and that the adrenal medulla has its own feedback system of orexin. In cultured pig adrenomedullary cells, it was shown that orexin A increased noradrenaline and adrenaline secretion [10]. As for the human adrenal medulla, a study using a human pheochromocytoma revealed that both orexin A and orexin B enhanced noradrenaline and adrenaline release via OX2R coupled to PLC-PKC signaling [25]. Despite the differences among species, there has been an accumulation of evidence indicating that the orexin system regulates catecholamine synthesis and release through common intracellular signaling.

It has been demonstrated that the orexin system has a pivotal role in the control of the autonomic nervous system. Shirasaka et al. revealed that the intracerebroventricular administration of orexin elevated plasma noradrenaline and adrenaline levels, blood pressure, heart rate and renal sympathetic nerve activity [26], suggesting that orexin activates both the sympathetic outflow and the sympatho–adrenomedullary system. Moreover, the OX1R-selective antagonism attenuated the elevation of plasma noradrenaline and blood pressure induced by orexin A [27]. Of note, an in vivo study demonstrated that orexin knockout mice experienced 10–15 mmHg lower blood pressure than wild-type mice [28]. These results suggest that the orexin system is physiologically involved in controlling the autonomic nervous system, and the investigation of the roles of orexin in the adrenal medulla from the viewpoint of the sympatho–adrenomedullary system is essential.

Although we focused on the roles of BMP-4 in this study, other BMP ligands also have functional roles in adrenomedullary cells, as previously mentioned. As well as BMP-4, BMP-2, BMP-7 and activin were expressed in PC12 cells and reduced the secretion of cAMP and dopamine [15]. Furthermore, BMP-2 and BMP-4 reduced DNA synthesis in a dose-dependent manner by themselves, and BMP-7 and activin A suppressed DNA synthesis in the presence of dexamethasone [15]. It was also reported that BMP-2 enhanced fibroblast growth factor (FGF)-induced cell differentiation through the activation of Smad and the upregulation of FGF receptor-1 expression in PC12 cells [29,30]; interestingly, a selective inhibitor of BMP signaling, dorsomorphin, also induced neurite outgrowth via the activation of protein kinase A (PKA)-dependent ERK1/2 signaling in PC12 cells [31]. These results indicate that various BMP ligands expressed in the adrenal medulla, in addition to BMP-4, have regulatory roles in cell functions and might interact with orexin, which should be examined in a future study.

Recently, it was proposed that the cortex and the medulla functionally interact in a paracrine fashion in the adrenal gland, not as two independent endocrine systems. They appear to be interwoven and show multiple contact zones without separation via connective tissue or interstitial membranes [32]. Dopamine secreted from the adrenal medulla suppresses aldosterone secreted from the adrenal cortex via the D2 dopamine receptor [33]. Conversely, endogenous glucocorticoids stimulate catecholamine synthesis by inducing the expression of enzymes in the adrenal medulla [34]. We found in previous studies that endogenous BMP-4 and melatonin also regulate catecholamine synthesis under the influence of adrenocortical steroids [16,19]. Of interest, we also reported that the BMP system and orexin regulate adrenocortical steroidogenesis by interacting with each other [17]. Considering that the BMP system and orexin regulate catecholamine synthesis, as shown in the present study (Figure 2 and Figure 3), orexin might be a key modulator between cortical and medullar tissues in the whole adrenal.

In the present study, there were several limitations, as with all in vitro experiments. First, the protein levels of synthetic enzymes and catecholamine were not examined since we aimed to evaluate the interactions of intracellular signaling between orexin and BMP in this study. It was reported that both orexin A and BMP-4 reduced the secretion of dopamine [13,15], and it was expected that co-treatment with orexin A and BMP-4 would further reduce the levels of dopamine, as speculated from the reduced mRNA levels of Th. Next, the data obtained in this study are limited to the rat pheochromocytoma PC12 cell line. Considering that the effects of orexin depend on cell lines and species, as mentioned above, the interaction between orexin and BMP signaling should be examined through in vivo studies using various animal species.

## 4. Materials and Methods

### 4.1. Experimental Reagents

Dulbecco’s Modified Eagle Medium (DMEM), fetal bovine serum (FBS) and penicillin/streptomycin solution (PS) were purchased from Sigma-Aldrich Co., Ltd. (St. Louis, MO, USA). Horse serum (HS) was purchased from Thermo Fisher Scientific Inc. (Waltham, MA, USA). Recombinant human BMP-4 was obtained from R&D Systems Inc. (Minneapolis, MN, USA), and human orexin A was obtained from Wako Pure Chemical Industries, Ltd. (Osaka, Japan). The PC12 rat pheochromocytoma cell line was obtained from RIKEN (Saitama, Japan) and was cultured in DMEM supplemented with 10% FBS, 10% HS and 1% PS with 5% CO_2_ at 37 °C. The cell cultures were passaged at nearly 80% confluence.

### 4.2. Quantitative Real-Time PCR Analysis

PC12 cells (3 × 10^5^ cells/mL) were treated with orexin A (10 to 300 nM) and BMP-4 (3 or 30 ng/mL) in DMEM containing 1% FBS, 1% HS and 1% PS in 12-well plates for 24 h. Total cellular RNAs were extracted using the TRI Reagent^®^ (Cosmo Bio Co., Ltd., Tokyo, Japan), and RNA concentrations were measured using the NanoDrop^TM^ One spectrophotometer (Thermo Fisher Scientific). The extracted RNA was subjected to reverse transcription using ReverTra Ace^®^ (TOYOBO Co., Ltd., Osaka, Japan). Primer pairs for target genes were selected from different exons to avoid amplifying PCR products derived from genomic DNA. Primer pairs for Th, 3,4-dihydroxyphenylalanie (DOPA) decarboxylase (Ddc), dopamine β-hydroxylase (Dbh), Smad6, Smad7, activin receptor-like kinase (ALK)-3 and ribosomal protein L19 (RPL19) as a housekeeping gene for PC12 cells [35] were utilized, as reported earlier [15,16,19]. Other primer pairs were prepared as follows: OX1R, forward-CGTGGCCGTGTTTCTCATAG and reverse-GCCATGATTCGGTGATGTCC (from GenBank accession #NM_013064.2); OX2R, forward-AACTGGTCATCTGCTTCGGA and reverse-ACTGTCCTCATGTGGTGGTT (NM_013074.2); ALK-2, forward-TGTCTGTGTGGATCAACAGAGG and reverse-TGGGTTCTGGTACCAGCATTC (NM_024486.1); BMP type-II receptor (BMPRII), forward-GACAACATTGCCCGCTTTAT and reverse-ATCTCGATGGGAAATTGCAG (NM_080407.1). After optimizing the annealing conditions [19], a quantitative PCR (qPCR) analysis was performed using the LightCycler^®^ 96 system (Roche Diagnostic Co., Tokyo, Japan). The relative expression of mRNA levels of the target genes was calculated using the Δ threshold cycle (Ct) method. ΔCt is the value obtained via subtracting the Ct values of RPL19 from those of the target genes. The expression level of target mRNA relative to RPL19 mRNA was expressed as 2^−(ΔΔCt)^. The data are shown as ratios of target mRNA to RPL19 mRNA.

### 4.3. Western Immunoblotting Analysis

PC12 cells (3 × 10^5^ cells/mL) were pretreated with orexin A (300 nM) in DMEM containing no serum and 1% PS for 48 h. After a 1 h stimulation with BMP-4 (3 ng/mL), the cells were solubilized via a sonicator in a 100 μL RIPA lysis buffer (Upstate Biotechnology, Lake Placid, NY, USA) containing 1 mM of Na_3_VO_4_, 1 mM of NaF, 2% SDS and 4% β-mercaptoethanol as reported previously [19]. The samples were then subjected to SDS-PAGE/immunoblotting analysis with primary antibodies, anti-phospho-Smad1/5/9 (pSmad1/5/9) antibodies (13,820, Cell Signaling Technology, Inc., Beverly, MA, USA) and anti-total-Smad1 (tSmad1) antibodies (9743, Cell Signaling Technology, Inc.), at 1:500 dilution and then with anti-IgG antibodies (7074, Cell Signaling Technology, Inc.) at 1:10^4^ dilution. The integrated band intensities were measured using the C-DiGit^®^ Blot Scanner System (LI-COR Biosciences, Lincoln, NE, USA). Phospho-Smad1/5/9 levels were evaluated using the ratios of the signal intensities of phospho-Smad1/5/9/total-Smad1.

### 4.4. Statistics

All data were obtained from at least three separate experiments performed with triplicate samples. All results are shown as the means ± standard errors of the mean (SEM). Statistical analysis was performed using an ANOVA or the unpaired *t*-test. *p* values < 0.05 were shown to be statistically significant.

## 5. Conclusions

The results of this study indicate that both orexin and BMP-4 suppress the expression of rate-limiting enzymes during catecholamine biosynthesis. In this mechanism, orexin and BMP-4 mutually enhance the intracellular signaling of each other by suppressing the expression of inhibitory Smad6/7, upregulating the expression of BMPRII via orexin A and upregulating the expression of OX1R via BMP-4. Further investigation on the functional inter-relationship between orexin and the BMP system in the adrenal medulla is key to expanding our knowledge about the sympatho–adrenomedullary system and cortical–medullary crosstalk.

## Data Availability

The data presented in this study are available on request from the corresponding author.

## Abbreviations

ACTH, adrenocorticotropin; ALK, activin receptor-like kinase; BMP, bone morphogenetic protein; BMPRII, BMP type-II receptor; Dbh, dopamine β-hydroxylase; Ddc, 3,4-dihydroxyphenylalanie decarboxylase; DOPA, 3,4-dihydroxyphenylalanie; FGF, fibroblast growth factor; HPA, hypothalamic–pituitary–adrenal; OXA, orexin A; OX1R, orexin receptor type 1; OX2R, orexin receptor type 2; PKA, protein kinase A; PKC, protein kinase C; RPL19, ribosomal protein L19; TGF, transforming growth factor; and Th, tyrosine hydroxylase.
